# Solar thermochemical CO_2_ splitting with doped perovskite LaCo_0.7_Zr_0.3_O_3_: thermodynamic performance and solar-to-fuel efficiency

**DOI:** 10.1039/d0ra05709f

**Published:** 2020-09-29

**Authors:** Lei Wang, Tianzeng Ma, Shaomeng Dai, Ting Ren, Zheshao Chang, Mingkai Fu, Xin Li, Yong Li

**Affiliations:** Institute of Electrical Engineering, Chinese Academy of Sciences Beijing 100190 China fumingkai@mail.iee.ac.cn; University of Chinese Academy of Sciences Beijing 100190 China; School of Mechanical Engineering, University of Science and Technology Beijing Beijing 100083 China liyong@ustb.edu.cn

## Abstract

The research of thermochemical CO_2_ splitting based on perovskites is a promising approach to green energy development. Performance evaluation was performed towards the doped perovskite LaCo_0.7_Zr_0.3_O_3_ (LCZ-73) based two-step thermochemical CO_2_ splitting process thermodynamically based on the experimentally derived parameters for the first time. The impacts of vacuum pump and inert gas purge to reduce oxygen partial pressure and CO_2_ heating on the performance parameter *η*_solar-to-fuel_ have been analyzed. The results showed that at the *P*_O_2__ of 10^−5^ bar, non-stoichiometric oxygen *δ* increased by more than 3 times as the reduction temperature varied from 1000 °C to 1300 °C, however, no significant deviation of *δ* was observed between 1300 °C and 1400 °C. The reaction enthalpy ranged from 60 to 130 kJ mol^−1^ corresponding to *δ* = 0.05–0.40. Comparing the abovementioned two ways to reduce the oxygen partial pressure, the *η*_solar-to-fuel_ of 0.39% and 0.1% can be achieved with 75% and without heat recovery with the CO_2_ flow rate of 40 sccm under experimental conditions, respectively. The energy cost for CO_2_ heating during the thermodynamic process as the *n*_CO_2__/*n*_LCZ-73_ increases was obtained from the perspective of energy analysis. The ratio of *n*_CO_2__/*n*_LCZ-73_ at lower temperature required more demanding conditions for the aim of commercialization. Finally, the ability of perovskite to split CO_2_ and thermochemical performance were tested under different CO_2_ flow rates. The results showed that high CO_2_ flow rate was conducive to the production of CO, but at the cost of low *η*_solar-to-fuel_. The maximum solar-to-fuel efficiency of 1.36% was achieved experimentally at a CO_2_ flow rate of 10 sccm in the oxidation step and 75% heat recovery.

## Abstract 2

Thermodynamics analysis of two-step thermochemical CO_2_ splitting with LaCo_0.7_Zr_0.3_O_3_ with gas–gas, gas–solid phase heat recuperation is performed based on experiment.

## Graphical abstract

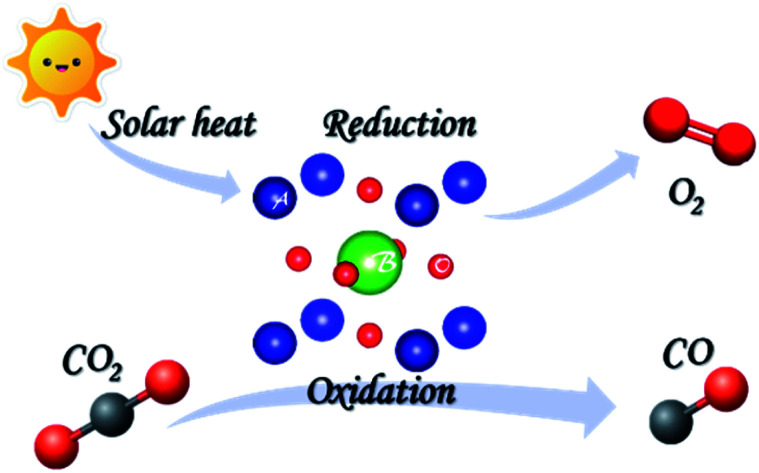

## 1. Introduction

CO_2_ is the main greenhouse gas of the Earth because of its stable thermodynamic properties and large amounts of emission in a short period of time.^1^ Thermochemical CO_2_ splitting is an effective method to mitigate CO_2_ emissions and provide a sustainable energy development path.^2^ As the most abundant energy source on the Earth, solar energy is clean and pollution-free, and has received the attention of many countries in the world. Using solar energy as a source of energy for thermochemical fuel production can effectively alleviate the global energy crisis and global warming effect.^3,4^ Solar thermochemical CO_2_ splitting takes sufficient solar energy as the heat source and greenhouse gas CO_2_ as the carbon source, which can realize the sustainable production of carbonaceous fuel,^5,6^ and can be converted into liquid fuels through existing industrial facilities for long-distance transportation and storage.^7^ At the same time, in this process, discontinuous and low energy density solar energy is stored in the form of fuel chemical energy with high energy density.

The solar two-step thermochemical fuel production with metal oxide perovskite, ABO_3_, as the oxygen carrier has received more and more attention from the researchers in recent years.^8,9^ Compared to the more studied binary oxide cerium oxide, perovskites have the advantage of being able to be reduced at lower operating temperatures, while having a greater amount of oxygen released so that more fuel can be produced in the oxidation step.^10,11^ In recent years, it has been reported that perovskite is used as oxygen carrier to split CO_2_ for CO production.^12,13^ Dey *et al.* studied the substituted La_0.5_Sr_0.5_MnO_3_ with trivalent metal ion at B site, and the results showed that the performance of thermochemical CO_2_ splitting was significantly improved by 5% Sc substitution at B position, and the yield of O_2_ and CO increased by 2 times and 1.7 times, respectively.^14^ Muhich *et al.* compared the performance of perovskite and ceria in thermochemical splitting of CO_2_, and found that perovskite has a high CO production capacity under a large CO_2_ flow rate, but at the same time, the solar-to-fuel efficiency is lower due to the high heating cost.^15^ Ezbiri *et al.* extensively screened the perovskite redox activity and thermochemical stability used for solar thermochemical splitting of CO_2_ by density functional theory, and verified the calculation results through experiments and applied them to fine-tune the oxygen exchange capacity of selected perovskite components.^16^ Galvez and coworkers studied the splitting of CO_2_ by Ca and Sr-doped LaMnO_3_ perovskites and found that the formation of carbonates resulted in a decrease in thermochemical efficiency due to incomplete conversion of CO_2_.^17^ Demont *et al.* studied the solar CO_2_ splitting *via* two-step thermochemical cycle with A- and B-site substituted Mn-perovskites, the results showed the effectiveness of the incorporation of Y in A-site and Mg in B-site in terms of CO production when compared with ceria.^18^ Cooper *et al.* studied LaMnO_3_ perovskite with Ca/Sr A-site and Al B-site doping for CO_2_ splitting, the results show that these materials could split CO_2_ into CO at 1240 °C and could approach complete oxidation at 1040 °C. The fuel production per unit mass was 10 times that of CeO_2_ under the same conditions.^19^ Bork and coworkers found a material, La_0.6_Sr_0.4_Cr_0.8_Co_0.2_O_3−*δ*_ with efficient solar-to-fuel conversion at lower temperatures. Thermogravimetric experiments showed that the ability to decompose CO_2_ is more than 25 times that of CeO_2_ in the two-step thermochemical cycle of 800–1200 °C.^20^ Demont *et al.* studied the splitting of CO_2_ by two-step solar thermochemical of LaMnO_3_ perovskite doped with Sr, and characterized the redox thermodynamics properties. The results showed that a certain amount of Sr can tune the redox thermodynamics of the series of materials, which has a high activity for the splitting of CO_2_.^21^ Takalkar *et al.* studied the thermochemical CO_2_ splitting by Pr_*x*_Sr_1−*x*_MnO_3_ perovskites by thermogravimetry. It was found that lower Pr and higher Sr can effectively increase CO production, and two optimal perovskites were identified as promising materials for thermochemical CO_2_ splitting.^22^ Jiang *et al.* studied the loaded LaFeO_3_ perovskite with A- and B-sites doping. The CO production increased by 2–3 times, and the controlling mechanism and kinetic model of CO_2_ splitting process were analyzed.^23^

Currently, in the two-step thermochemical CO_2_ splitting with perovskite as the oxygen carrier, most of the researches focus on one or the perovskite series through the different contents of elemental doping. The main goal of the studies is to find a high-performance perovskite that increases fuel production and thus achieves high solar-to-fuel efficiency (*η*_solar-to-fuel_). The direction of efforts is mainly focused on the improvement of redox thermodynamics, kinetic properties and thermal stability of the material thermochemical cycle. Although researchers have made a lot of efforts in these areas and made great progress in some areas, the current researches in the field of thermochemical fuel research have low efficiency and cannot be commercialized. At present, the world's largest thermochemical fuel system is in pilot scale,^24^ and how to achieve efficient fuel production remains a major challenge. It is beneficial to explore materials that can balance the advantages of reduction and oxidation reactions, and thermodynamic analysis should be performed when conducting primary materials screening.^25^ However, there are few studies on the thermodynamics of perovskite thermochemical fuel production. Thermodynamic analysis was only performed theoretically in literatures. Carrillo and Scheffe established a thermodynamic model to calculate the solar-to-fuel efficiency of La_1−*x*_(Sr,Ca)_*x*_Mn_1−*y*_Al_*y*_O_3_ perovskites, showing the potential to improve the efficiency during isothermal or near-isothermal thermochemical cycles.^26^ Muhich *et al.* implemented a thermodynamic system model of solar thermochemical fuel production based on mass and energy balances, the results showed the promising of perovskites due to the high fuel production capacities under large H_2_O/CO_2_ flow rates, but the poor solar-to-fuel efficiency due to the high heating load.^15^ Bork *et al.* performed a Calphad defect model to optimize and calculate the redox thermodynamics of Cr-doped La_0.6_Sr_0.4_MnO_3_ perovskite for solar-to-fuel conversion, the results revealed that both the thermochemical fuel production and the solar-to-fuel efficiency of the Cr-doped materials were improved. These predictive perspectives are expected to provide prospects for the thermodynamic engineering of perovskite thermochemical reactions towards high solar-to-fuel efficiency.^27^ Overall, the existing studies on thermodynamic analysis and solar-to-fuel efficiency mainly focus on theoretical analysis, but the extent to which level of this efficiency, especially the solar-to-fuel efficiency still needs more experimental proof.

To our knowledge, there are no studies addressing the thermodynamic analysis of CO_2_ conversion into fuels using Zr doped LaCoO_3_. This paper focuses on the report of a detailed thermodynamic analysis of a two-step thermochemical CO_2_ splitting process with perovskite LaCo_0.7_Zr_0.3_O_3_ (LCZ-73) as the oxygen carrier based on the obtained thermodynamic parameters experimentally. Material synthesis, characterization and thermochemical performance of CO_2_ splitting have been reported in the previous experimental work,^28^ in which studies of elements doping, doping concentration, the redox temperatures, heating rates on the performance of the doped LaCoO_3_ perovskites based two-step thermochemical CO_2_ splitting and reduction kinetics were illustrated. The results demonstrated the stability of the material before and after the thermochemical redox cycles, the activity of thermochemical CO_2_ splitting and better reduction kinetics than other perovskite metal oxides and cerium oxides reported in the literature. These studies focused on discussing the performance of thermochemical CO_2_ splitting from the perspective of experiments, but did not involve the thermodynamics of this process. Therefore, this paper discussed the thermodynamics of thermochemical CO_2_ splitting from the perspective of solar to fuel conversion. The thermochemical reduction enthalpy was obtained and the thermodynamic efficiency was analyzed with the consideration of gas–gas, gas–solid phase heat recuperation as well as the use of vacuum pump and vacuum pump combined with inert gas purge to reduce the oxygen partial pressure. Based on the experimental conditions, the solar-to-fuel efficiency of the solar thermochemical fuel production process was analyzed by combining the vacuum pump with the inert gas purge and directly the vacuum pump to reduce the oxygen partial pressure, respectively. The energy consumed for CO_2_ heating took up the bulk of energy consumption of the thermodynamic process as the *n*_CO_2__/*n*_LCZ-73_ increases based on the energy analysis. Finally, the thermodynamic model was verified by experimentally testing the CO_2_ splitting performance under different CO_2_ flow rates and analyzing its solar-to-fuel efficiency. This paper revealed that one of the main problems in the field of solar thermochemical fuel production was the huge energy input caused by excessive oxidant in the oxidation step, which resulted in low efficiency.

## 2. Experiment and mathematical model

### 2.1. Experimental procedure and apparatus

**Fig. 1:**
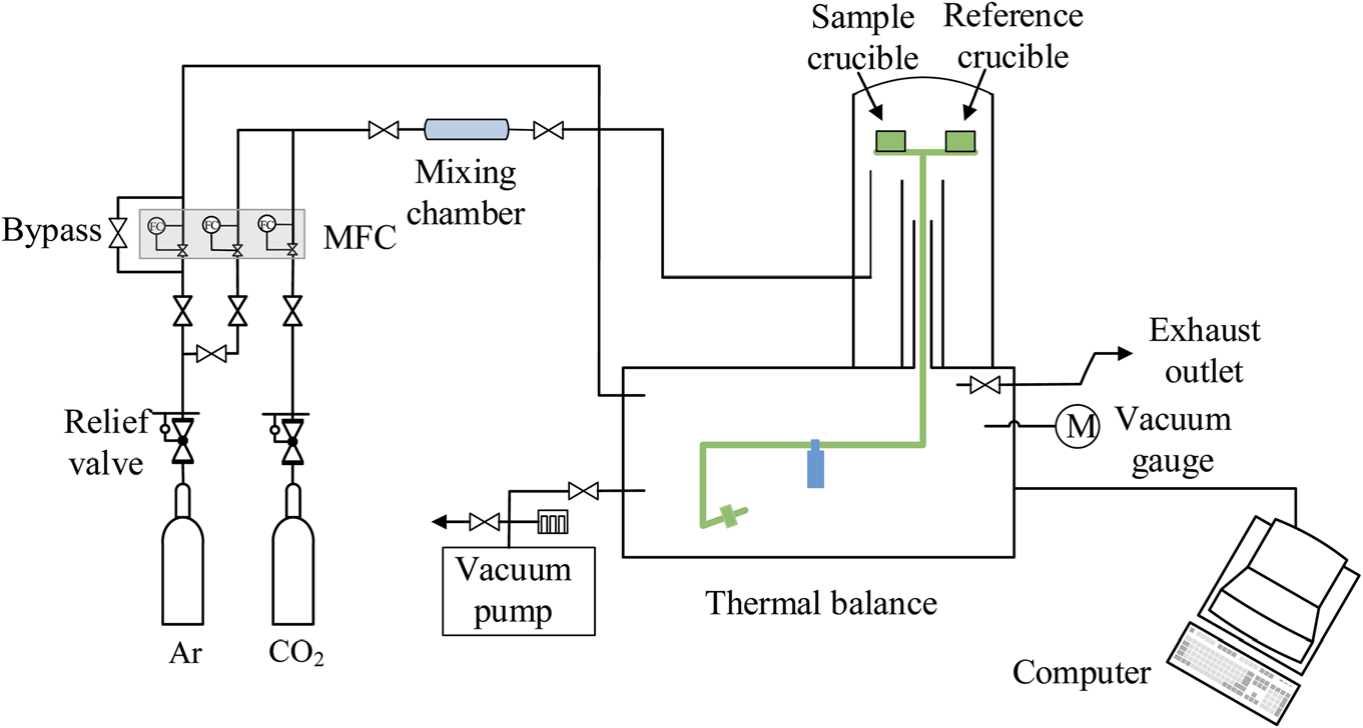
Schematic diagram of the experimental platform for thermochemical CO_2_ splitting.

LaCo_0.7_Zr_0.3_O_3_ perovskite powder was prepared by citric acid sol–gel method, which was described in detail in previous studies.^28^ Metal nitrate precursors were obtained commercially (Aladdin and Macklin) and were specified to be >99.5% pure. XRD measurements confirmed the existence of perovskite structure LaCo_0.7_Zr_0.3_O_3_, SEM image showed that the sample was irregularly shaped particles with a size in the a few microns and EDS analysis verified that the synthesized sample compositions were in line with expectations. Similarly, XRD of the powder sample after reduction showed that it still maintained the perovskite phase structure. The measurements of the redox reactions were carried out with a high temperature thermogravimetric analyzer (TGA, LINSEIS STA PT 1600). To avoid the effects of thermal inertia during testing, fresh material of LaCo_0.7_Zr_0.3_O_3_ (LCZ-73) oxide of the same batch of synthetic sample powder was used in each test. Granular powders with an average weight of approximately 10 mg were evenly placed in an alumina crucible with a volume of about 100 μl throughout all the measurement series. Before the formal start of the redox experiments, a vacuum pump was used to vacuum the furnace chamber to 5.5 × 10^−5^ atm for a low oxygen environment, and then purged with high purity argon (99.999%) for one hour to provide an inert environment and further reduce the oxygen partial pressure. The inert atmosphere was always maintained throughout the reduction process and during the process of reducing from reduction temperature to the oxidation temperature to blow the released oxygen out of the furnace in time. High purity argon was used as the purge and protective gas, the same high purity carbon dioxide (99.999%) was used as the oxidant during the oxidation process. The overall gas flow rate entering the reaction chamber was maintained at 80 sccm, with the balance being protected with argon flow of 40 sccm at all times. In the reduction process, the sample was heated to 1000, 1100, 1200, 1300, 1400 °C, respectively, at a heating rate of 30 °C min^−1^ and maintained for 60 min with continuous and constant Ar flow. Then the temperature was lowered to 800 °C at the same rate for oxidation. In the oxidation step, the high purity Ar (40 sccm) and high purity CO_2_ with a flow rate of 40 sccm (*P*_CO_2__ = 0.5 atm) were mixed and introduced into the furnace chamber to react with the oxygen-deficient perovskite and produce CO. The trace oxygen was blown out and diluted to further reduce the impact on CO_2_ splitting. This oxidation process proceeded for 60 min. The specific experimental platform was shown in the Fig. 1.

Since the redox behavior analysis of perovskite in this work was mainly based on thermogravimetric analysis, the mass change in sample was induced by the release and uptake of oxygen under high temperatures and specific oxygen partial conditions, then the non-stoichiometry of the perovskite can be determined by measuring the mass change. The relationship between the mass change Δ*m* and the nonstoichiometric oxygen *δ* is as follow:^29^1
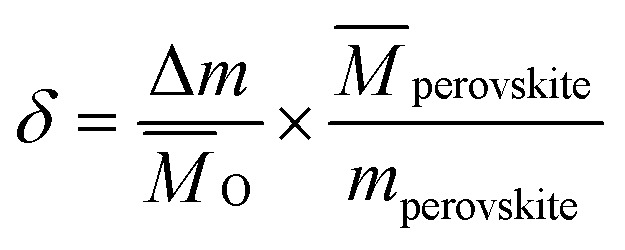
*m*_perovskite_ is the initial mass of the perovskite sample, and *M̄*_perovskite_ and *M̄*_O_ are the molar mass of the perovskite and the atomic oxygen, respectively.

Thermodynamic parameters are extracted from the Van't Hoff approach. The standard molar Gibbs free energy of the reduction reaction can be expressed as follow:^8,30^

2 Δ*G*^Θ^(*T*) = −*R̄T* ln *K*_red_ = Δ*H*^Θ^_red_ − *T*Δ*S*^Θ^_red_

where *K*_red_ is the equilibrium constant, and *K*_red_ = *P*_O_2__/*P*^Θ^. *P*_O_2__, *P*^Θ^, *R̄* and *T* are the oxygen partial pressure, the standard pressure and *P*^Θ^ = 1 bar, the universal gas constant and the reduction temperature, respectively. Inserting the relationship of *K*_red_ into eqn (1), for constant oxygen non-stoichiometry, *δ*, the above equation can be rearranged as3
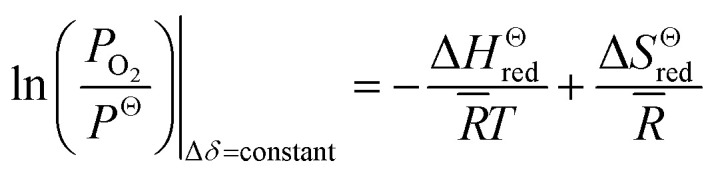
Δ*H̄*^Θ^_red_ is the standard molar enthalpy and can be determined through an Arrhenius plot for sets of temperature *T* and ln(*P*_O_2__).

### 2.2. Mathematical model

A typical thermochemical redox cycle of CO_2_ splitting for a two-step process based on perovskite can be exemplified in the following reactions:4
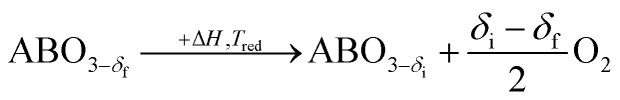
5
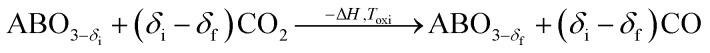
where *δ*_i_ and *δ*_f_ are the oxygen non-stoichiometry of perovskite after reduction and oxidation reactions, respectively. Δ*δ* = *δ*_i_ − *δ*_f_ is the non-stoichiometry net change in oxygen, which is also a numerical equivalent of the amount of CO produced by 1 molar of perovskite in a single thermochemical cycle. In the first step, the perovskite oxide is partly reduced at a high reduction temperature (*T*_red_), releasing lattice oxygen and generating oxygen vacancies, eqn (4). In the second oxidation step, the reduced perovskite is reoxidized by CO_2_ at a lower temperature (*T*_oxi_), eqn (5), CO_2_ is reduced to CO. For a non-isothermal thermochemical cycle, *T*_red_ > *T*_oxi_.

#### 2.2.1. Reduction process

Here we consider a dynamic thermochemical cycle rather than a process of heating the perovskite from room temperature to reduction temperature, and a quasi-steady state is assumed for the thermochemical cycle. Thus, the energy required to heat the perovskite from oxidation temperature to reduction temperature is:6
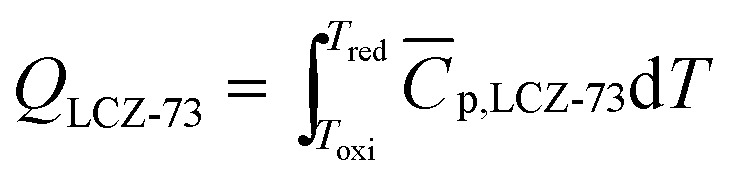


The energy required to drive the reduction reaction of one molar perovskite is:7
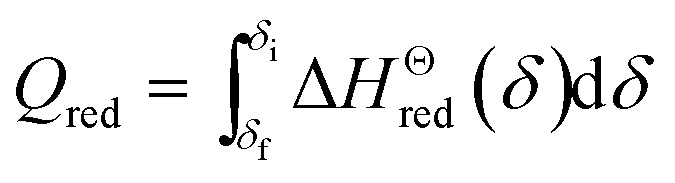
where *C̄*_p,LCZ-73_ is the molar specific heat capacity of perovskite LCZ-73 used in the test, which can be obtained by differential scanning calorimeter. According to the thermal effect obtained by differential scanning calorimetry and the formula Δ*Q* = (*m*/*M̄*)*C̄*_p_Δ*T*, the value of *C̄*_p_ can be obtained. In fact, the specific heat capacity is a function of temperature, but for convenience, the average value of *C̄*_p,LCZ-73_ = 157.5 J (mol^−1^ K^−1^) is considered.

Researches have shown that decreasing oxygen partial pressure in the thermal reduction process is beneficial to the increase of *δ* and the efficiency of thermochemical fuel production.^31^ There are two common ways to reduce oxygen partial pressure of the reduction process in thermochemical fuel research: vacuum pump and inert gas purge.

##### 2.2.1.1. Pumping

A vacuum pump is needed to eliminate the residual air in the reactor to provide a lower oxygen partial pressure environment during the thermal reduction process. *Q*_pump_ is the energy consumed to obtain a lower oxygen partial pressure with a vacuum pump. In this paper, the ideal pump work is used to calculate the performance of solar-to-fuel efficiency.^32^

8 *Q*_pump_ = *n*_O_2__*R̄T*_pump_ ln(*P*_0_/*P*_O_2__)/*η*_S→W_

where *n*_O_2__ = *n*_CO_/2 follows from the reaction stoichiometry, *R̄* is the universal gas constant, *T*_pump_ is the operating temperature of the pump. In this study, the temperature is the ambient temperature. *P*_0_ is the experimental pressure at atmospheric pressure. *P*_O_2__ is the oxygen partial pressure inside the instrument after vacuuming, *η*_S→W_ is the solar energy to pump work conversion efficiency and its value is assumed to be 0.1 in this paper.^32^

##### 2.2.1.2. Sweep gas

Argon was used as the sweep gas in the reduction step and as the shielding gas for the balance chamber throughout the thermochemical process. In our experiment, high purity Ar (99.999%) with an oxygen impurity concentration less than 10 ppm was used. Considering that the high-temperature exhaust gas in the reduction process carries a large amount of heat, it can be used to preheat Ar required for the reduction reaction. Therefore, the energy required to heat sweep gas by considering the heat recovery of exhaust gas is calculated as:9
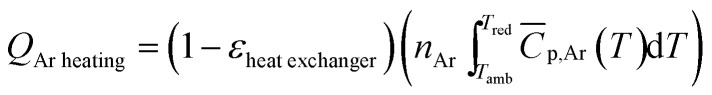
where *ε*_heat exchanger_ is the heat exchange coefficient of the heat exchanger.^33^*T*_amb_ is the ambient temperature and *T*_red_ is the reduction temperature. The molar specific heat capacity of Ar is considered constant at a certain temperature (*T* ≤ 2000 K), and *C̄*_p,Ar_ = 20.786 J (mol^−1^ K^−1^).^34^

#### 2.2.2. Oxidation process

Energy cost during the oxidation process is the cost of heating CO_2_. A heat exchanger can be used to heat CO_2_ using the outflowing oxidizer and fuel from the oxidation reactor. The same heat exchanger coefficient is used for simplicity.10
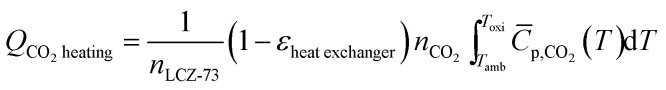

11 *C̄*_p,CO_2__(*T*) = 20.99 + 6.768 × 10^−2^*T* − 4.960 × 10^−5^*T*^2^ + 1.779 × 10^−8^*T*^3^ − 2.495 × 10^−12^*T*^4^

where *n*_CO_2__ is the molars of CO_2_ introduced in the oxidation process, *T*_oxi_ is the oxidation temperature, *C̄*_p,CO_2__ is the molar specific heat capacity of CO_2_ under a standard atmospheric pressure and is a function of temperature *T*, which can be given as a polynomial fits in J (mol^−1^ K^−1^).^33^

##### 2.2.2.1. Heat recovery

It is difficult to realize the heat utilization of solid reactants by heating low temperature solid with high temperature solid^35^ and may present some other problems. In many previous studies, the heat recovery of solid reactants was not considered.^33^ The idea of using a heat exchanger to convert solid heat into process heat to heat the oxidant to improve efficiency^33^ is also used in the analysis herein.

Under the experimental conditions, in order to maintain the oxidation reaction at a constant temperature, continuous additional heating is necessary. The heat transfer for one molar LCZ-73 during the oxidation reaction is calculated:

12 *Q*_oxi_ = −*Q*_red_ + (*δ*_i_ − *δ*_f_)HHV_CO_

The available amount of the recovered heat from the solid reactants is limited by the heat required for CO_2_ heating in the oxidation process,13
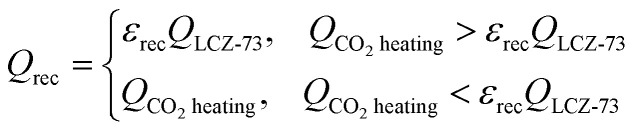
where *ε*_rec_ is the effectiveness of heat recovery. This assumes that *ε*_rec_ = 0.6 (ref. 33) and HHV_CO_ is the high calorific value of CO.

#### 2.2.3. Solar-to-fuel efficiency

An ideal blackbody reactor is considered in the thermochemical cycling reactions, therefore, the absorption efficiency of the reactor which is a function of *T*_red_ and concentration ratio *C*.^36,37^ Considering the radiative heat loss of the solar reactor, the heat required to power all the processes of the thermodynamic system can be supplied with the absorption efficiency *η*_abs_. It can be defined as14
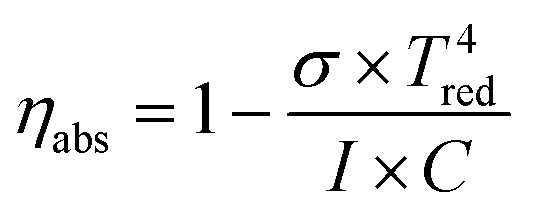
Here *σ* is the Stefan–Boltzmann constant, 5.67 × 10^−8^ W (m^−2^ K^−4^). *I* = 1000 W m^−2^, *C* represents the solar concentration ratio in suns, here, *C* = 3000 (ref. 38 and 39) is employed in this paper. Here, we assume that there is no heat loss of convection and heat conduction from the solar reactor, thus there is only the heat loss rooted in radiation to the ambient.

An energy balance equation for the entire thermochemical processes yields the following equation:^33,38^15
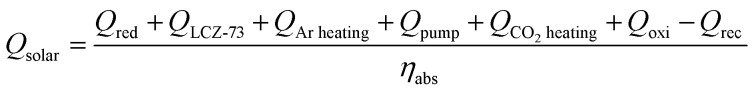


Here we assume that the required electrical heating energy, pumping power, energy for gas purging, CO_2_ heating and energy required to maintain a constant temperature for oxidation and other energy-consuming processes of the thermochemical cycle are all provided by solar energy. The available heat recovery of the system is also considered.

According to the first law of thermodynamic, *η*_solar-to-fuel_ of the two-step thermochemical redox processes is chosen as the merit of the thermochemical cycling performance,^40,41^ which is defined as the ratio of the calorific value of the fuel to the total solar energy input in the system:^33,38,42^16
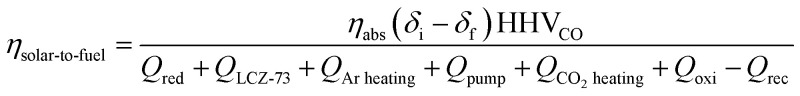
where HHV_CO_ is the higher heating value of CO.

## 3. Results and discussion

### 3.1. Thermodynamic characteristics of LCZ-73

**Fig. 2:**
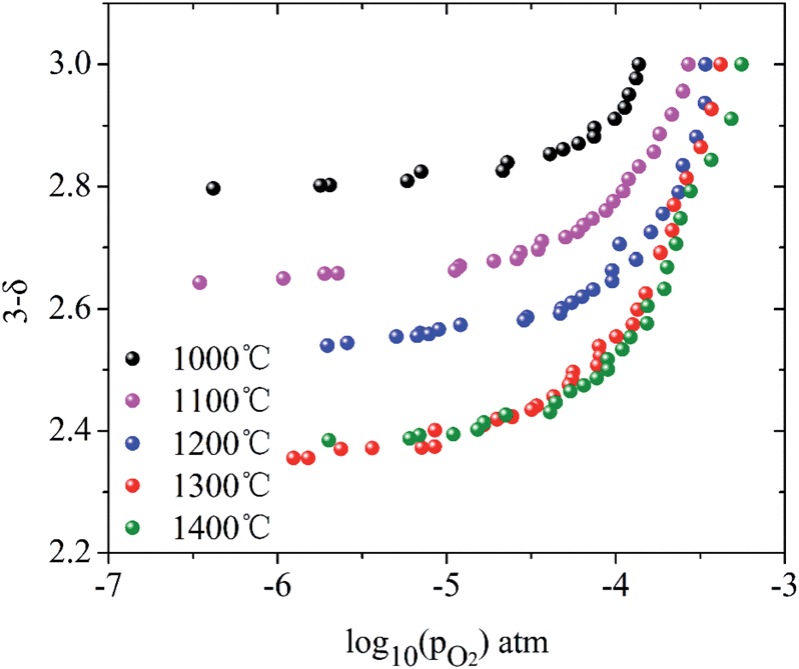
Oxygen non-stoichiometry *δ* of ∼10 mg LCZ-73 perovskite powder reduced at 1000–1400 °C for 60 min with a heating rate of 30 °C min^−1^ (in this analysis, only the reduction under a constant temperature process is considered).

Here, the relationship between oxygen partial pressure and non-stoichiometric oxygen *δ* during the reduction process after reaching the target temperatures is revealed. As shown in Fig. 2, the non-stoichiometric oxygen *δ* in LaCo_0.7_Zr_0.3_O_3−*δ*_ is highly dependent on reduction temperature and the oxygen partial pressure (*P*_O_2__). As the oxygen partial pressure (*P*_O_2__) decreases, the non-stoichiometric oxygen *δ* gradually increases, but the increase rate slows down as the reduction reaction proceeds. Under the same oxygen partial pressure environment, the high temperature is more favorable for the generation of non-stoichiometric oxygen in the reduction process. But it is not that the higher the temperature is, the better it will be. At 1300 °C and 1400 °C, the ability to produce non-stoichiometric oxygen under both conditions is nearly the same. At the oxygen partial pressure of 10^−5^ bar, the non-stoichiometric oxygen at the reduction temperature of 1000–1400 °C is 0.18, 0.34, 0.44, 0.60 and 0.60, respectively. The non-stoichiometric oxygen *δ* increases by more than 3 times when the temperature varies from 1000 °C to 1300 °C. At 1000 °C, the non-stoichiometric oxygen *δ* is doubled when *P*_O_2__ changes from 10^−4^ to 10^−5^ bar, while, at 1300 °C, this value increases by a third. Lower oxygen partial pressure is required when the same non-stoichiometric oxygen is reached at low temperature. At *δ* = 0.2, the oxygen partial pressure *P*_O_2__ during 1000–1400 °C varies from 10^−5.75^ to 10^−3.55^ bar.

**Fig. 3:**
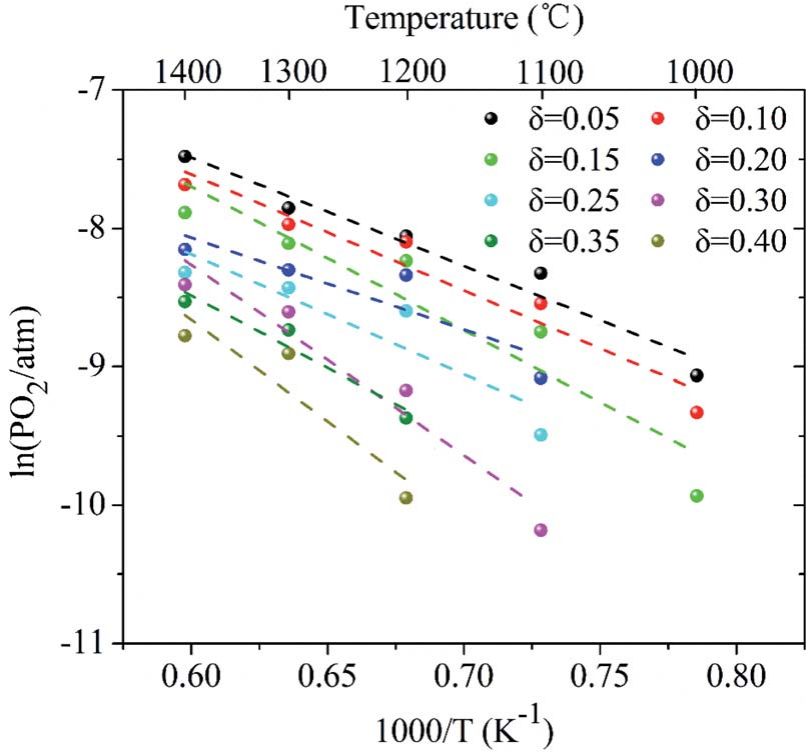
Arrhenius plots of oxygen partial pressure *versus* inverse temperature for LaCo_0.7_Zr_0.3_O_3_ (LCZ-73) corresponding to fixed *δ* between 0.05 and 0.40.

Based on the above analysis, the derived ln(*P*_O2_) *versus* inverse temperature for LCZ-73 is shown in Fig. 3. Considering that there is a certain error in the oxygen partial pressure at some points, the linearity is not ideal for all when performing linear fitting on the data points, especially for the *δ* = 0.20 and *δ* = 0.25. Similarly, it can be found that the non-stoichiometric oxygen increases with decreasing the oxygen partial pressure at the same temperature. In order to achieve the same *δ*, a lower oxygen partial pressure is required at low temperature. Fig. 3 also shows two ways to obtain larger non-stoichiometric oxygen *δ*: increasing temperature and decreasing oxygen partial pressure. According to the existing data with better linearity, it can be seen that Δ*H*^Θ^_red_ in eqn (3) is likely to be temperature-independent, which is confirmed in other studies.^30^ Simultaneously, this also shows the reliability of our experimental data. Based on this, we can get the relationship between the reduction enthalpy Δ*H*^Θ^_red_ and the different oxygen stoichiometry *δ* during the thermochemical reduction process, which will be discussed next.

**Fig. 4:**
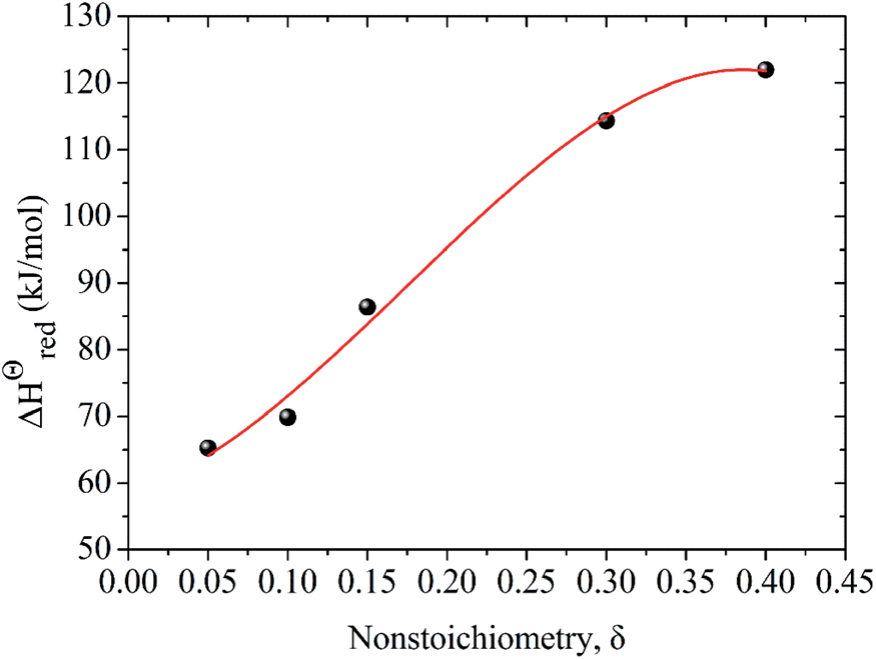
Variation of reduction enthalpy of LaCo_0.7_Zr_0.3_O_3_ (LCZ-73) as a function of oxygen non-stoichiometry *δ*.

The energy required to generate oxygen vacancies in the perovskite LCZ-73 can be obtained *via* the change of reduction enthalpy, Δ*H*^Θ^_red_. Similar studies have shown that reduction enthalpy is only related to stoichiometry *δ*.^8,43^ As shown in Fig. 4, the curve for the change in standard molar reduction enthalpy is fit with a polynomial. The value of Δ*H*^Θ^_red_ is 60–130 kJ mol^−1^ when *δ* in the range of 0.05–0.40. The following thermodynamic analysis will be based on these results for discussion.

17 Δ*H*^Θ^_red_(*δ*) = 58.529 + 71.905*δ* + 905.361*δ*^2^ − 1724.966*δ*^3^

### 3.2. Solar-to-fuel efficiency analysis for LCZ-73 based solar CO production system

#### 3.2.1. Decreasing *P*_O_2__*via* vacuum pump

**Fig. 5:**
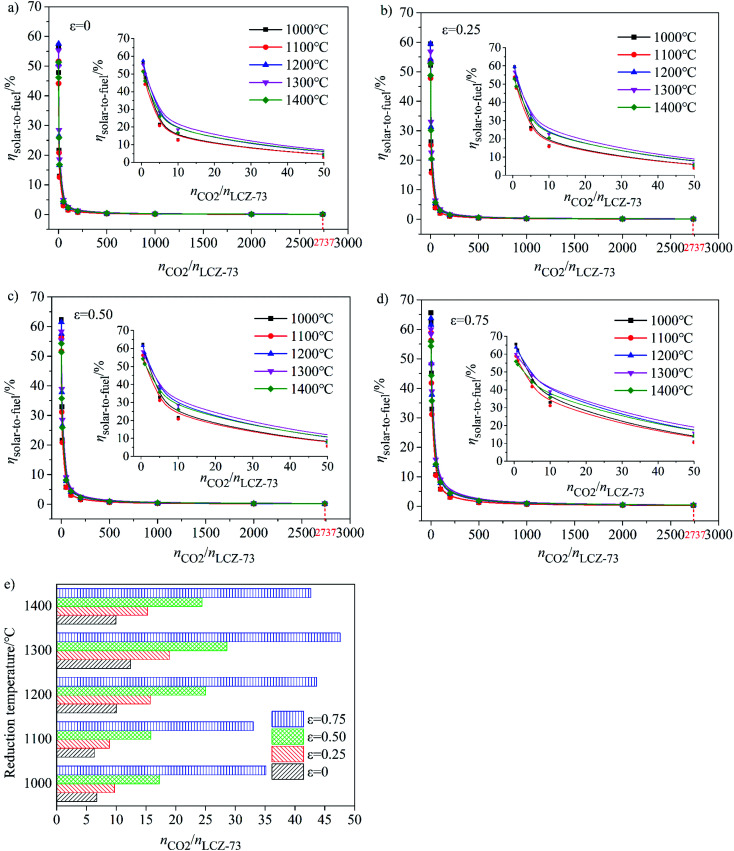
Solar-to-fuel efficiency (*η*_solar-to-fuel_) as functions of reduction temperature and molar ratio of CO_2_ to perovskite and the molar ratio of reactants required to achieve *η*_solar-to-fuel_ = 20% under different heat recovery conditions when using a vacuum pump to reduce oxygen partial pressure. (a)–(d) *η*_solar-to-fuel_ as functions of reduction temperature and molar ratio of CO_2_ to LCZ-73 under different heat exchange coefficients *ε*_heat exchanger_. (e) Molar ratio of CO_2_ to perovskite corresponding to *η*_solar-to-fuel_ = 20% with the reduction at 1000–1400 °C and oxidation at 800 °C.

In the case of decreasing the oxygen partial pressure by vacuum pump, then no sweep gas is needed and *Q*_Ar heating_ = 0. Solar-to-fuel efficiency of the two-step solar thermochemical CO_2_ splitting based on LCZ-73 perovskite as functions of reduction temperature and the ratio of *n*_CO_2__/*n*_LCZ-73_ with different heat exchange coefficients *ε*_heat exchanger_ is analyzed in the range of reduction temperature of 1000–1400 °C and oxidation at 800 °C and is shown in Fig. 5(a)–(d). Meanwhile, the relationship between the ratio of *n*_CO_2__/*n*_LCZ-73_ corresponding to *η*_solar-to-fuel_ = 20% and reduction temperatures is shown in Fig. 5(e).

As shown in Fig. 5(a)–(d), at the molar ratio of *n*_CO_2__/*n*_LCZ-73_ ≤ 500, *η*_solar-to-fuel_ decreases rapidly with the increase of the ratio, and decreases slowly when the molar ratio is greater than 500. In Fig. 5(a)–(c), *η*_solar-to-fuel_ ≤ 1% at *n*_CO_2__/*n*_LCZ-73_ ≥ 500, the molar ratio of *n*_CO_2__/*n*_LCZ-73_ is enlarged to 1000 in Fig. 5(d). This also means that higher efficiency can be achieved with higher heat recovery. This can also be found from Fig. 5(a)–(d) that with the increase of *ε*_heat exchanger_, *η*_solar-to-fuel_ increases gradually. In the current experimental situation, the molar ratio of *n*_CO_2__/*n*_LCZ-73_ is 2737 : 1, *η*_solar-to-fuel_ = 0.39% is obtained at the reduction temperature of 1300 °C with 75% heat recovery. While, when no heat recovery is considered, this efficiency is just 0.1%.

The insets are the partial enlargements with the molar ratio of less than 50. Overall, under the same conditions, the highest *η*_solar-to-fuel_ is achieved at 1300 °C and *n*_CO_2__/*n*_LCZ-73_ ≥ 5 in Fig. 5(a)–(c) and *n*_CO_2__/*n*_LCZ-73_ ≥ 10 in Fig. 5(d). When the ratio of *n*_CO_2__/*n*_LCZ-73_ < 10, *η*_solar-to-fuel_ will reach 20% or more in Fig. 5(a)–(c) and for higher heat recovery in Fig. 5(d), *η*_solar-to-fuel_ of 20% can be achieved at *n*_CO_2__/*n*_LCZ-73_ < 50. This efficiency value is also proposed based on estimated qualitatively in reference and is considered as the benchmark for commercially achievable system-level annual average solar-to-fuel efficiency for a viable alternative to conventional electrolysis.^44^ For this competitive efficiency reference value, further analysis is shown in Fig. 5(e). When the efficiency reaches 20%, *n*_CO_2__/*n*_LCZ-73_ = 47.59 at 1300 °C, and *n*_CO_2__/*n*_LCZ-73_ = 35.12 at 1000 °C with 75% heat recovery. The molar ratios of *n*_CO_2__/*n*_LCZ-73_ are 12.4 and 6.74 with no heat recovery, respectively. Compared with higher temperature conditions (1300 and 1400 °C), the molar ratio of *n*_CO_2__/*n*_LCZ-73_ at low temperature requires more demanding conditions with the prospects of commercialization. This is still a big challenge in the current research background of thermochemical fuels. However, under the same conditions, the larger molar ratio of *n*_CO_2__/*n*_LCZ-73_ is more accessible under current technical conditions.

#### 3.2.2. Decreasing *P*_O_2__*via* vacuum pump combined with Ar sweeping

**Fig. 6:**
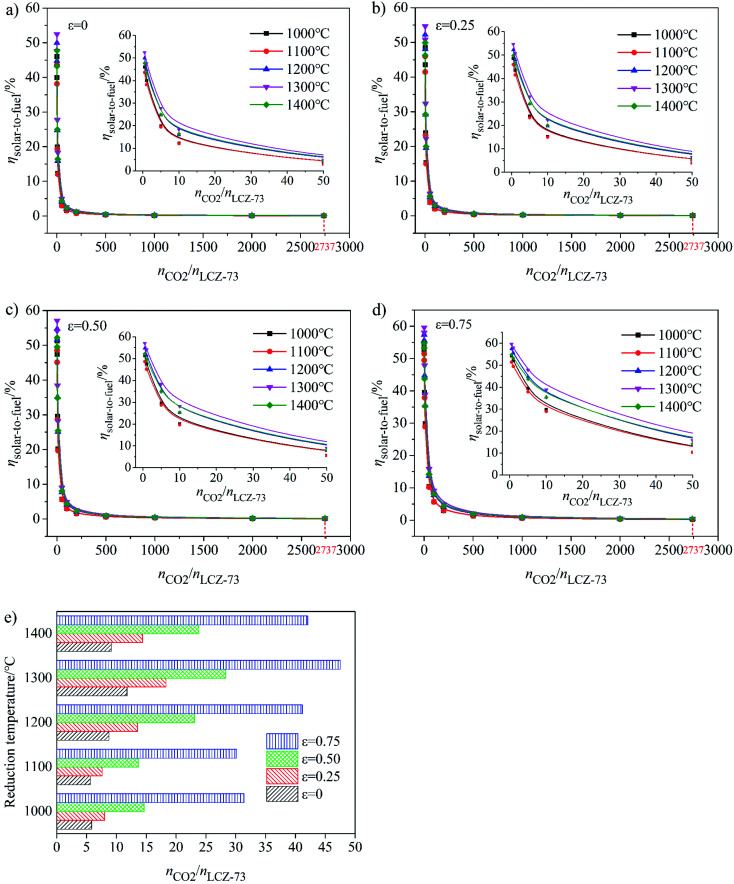
Solar-to-fuel efficiency (*η*_solar-to-fuel_) as functions of reduction temperature and molar ratio of CO_2_ to perovskite and the molar ratio of reactants required to achieve *η*_solar-to-fuel_ = 20% under different heat recovery conditions when using vacuum pump combined with gas purging to reduce oxygen partial pressure. (a)–(d) *η*_solar-to-fuel_ as functions of heat exchange coefficient *ε*_heat exchanger_ and molar ratio of CO_2_ to LCZ-73. (e) Variation of molar ratio of CO_2_ to LCZ-73 corresponding to *η*_solar-to-fuel_ = 20% with the reduction at 1000–1400 °C and oxidation at 800 °C.

When inert gas purging is used to reduce oxygen partial pressure, then no vacuum pump is needed and *Q*_pump_ = 0. A strategy of combined vacuum pump with Ar purging during the reduction process was adopted to reduce the partial pressure of oxygen in the reaction chamber rapidly and reduce the amount of Ar required for purging. It has also been shown that the combination of reducing the oxygen partial pressure *via* vacuum pump and inert sweeping helps to increase the efficiency of the inert-swept reactor.^31^ The evolution of *η*_solar-to-fuel_ as functions of reduction temperature and molar ratio of CO_2_ to LCZ-73 with different heat exchange coefficients *ε*_heat exchanger_ is depicted in Fig. 6(a)–(d). As shown, in the reduction temperature range of 1000–1400 °C, *η*_solar-to-fuel_ decreases rapidly as the molar ratio of CO_2_ to perovskite increases. When *n*_CO_2__/*n*_LCZ-73_ ≥ 1000, *η*_solar-to-fuel_ < 1%, regardless of the temperature is in this range, especially in Fig. 6(a)–(c), the range of this ratio expanded to *n*_CO_2__/*n*_LCZ-73_ ≥ 500. In Fig. 6(d), *ε*_heat exchanger_ = 0.75, *η*_solar-to-fuel_ > 1% at *n*_CO_2__/*n*_LCZ-73_ ≤ 500. When *n*_CO_2__/*n*_LCZ-73_ < 500, the efficiency slowly increases as the ratio decreases. For *n*_CO_2__/*n*_LCZ-73_ < 200, the efficiency increases rapidly as the ratio decreases. When it comes to the ratio of *n*_CO_2__/*n*_LCZ-73_ ≤ 200, *η*_solar-to-fuel_ > 1% except in the case of *ε*_heat exchanger_ = 0 and reduction temperatures of 1000–1100 °C. With the increase of *ε*_heat exchanger_, *η*_solar-to-fuel_ at different temperatures are improved and reached the highest at 1300 °C. Under the experimental conditions of this work, the flow rate of CO_2_ is 40 sccm, the molar ratio of *n*_CO_2__/*n*_LCZ-73_ = 2737, *η*_solar-to-fuel_ = 0.1% without considering heat recovery at the reduction temperature of 1300 °C, *η*_solar-to-fuel_ = 0.39% with 75% heat recovery. For a theoretical deduction, if the molar ratio of *n*_CO_2__/*n*_LCZ-73_ can be further reduced to 0.5, the theoretical maximum value of *η*_solar-to-fuel_ of 52.5% and 59.6% can be achieved with no heat recovery and 75% heat recovery were considered, respectively. Despite this considerable efficiency value, it is necessary to know that such a low ratio poses great challenges for experimental verification. The inset in each figure of Fig. 6(a)–(d) is for partial amplification in the range of molar ratio less than 50. It can be seen from the insets that the highest efficiency value is obtained over the entire molar ratio when the reduction temperature is 1300 °C, and the lowest value at 1000 °C. At this point we consider a considerable solar-to-fuel efficiency value of 20% which is a viable alternative with that more traditional and reliable technology based on electrolysis.^44^

Variation of molar ratio of *n*_CO_2__/*n*_LCZ-73_ corresponding to the solar-to-fuel efficiency of 20% with heat exchange coefficient *ε*_heat exchanger_ at different reduction temperatures was shown in Fig. 6(e). Similarly, we find that when the efficiency reaches 20% and as the benchmark, the ratio of *n*_CO_2__/*n*_LCZ-73_ is maximum at 1300 °C, reaching about 50, which is the easiest to reach in the temperature range in question. At the same time, this means that if there is a more efficient way to reduce the ratio of *n*_CO_2__/*n*_LCZ-73_, then at 1300 °C, solar-to-fuel efficiency will be higher than 20%. This is a great encouragement for commercialization. However, in most of the current experimental studies of two-step solar thermochemical fuel production cycles, the far excessive amount of oxidant in oxidation step is used for a high fuel production. How to effectively reduce the amount of oxidant introduced into the oxidation step becomes the key to improve the solar-to-fuel efficiency. Another finding is that as the heat exchange coefficient increases, *n*_CO_2__/*n*_LCZ-73_ at the same temperature changes monotonously, indicating that as the heat recovery increases, the conditions for commercialization are more moderate, and the solar-to-fuel efficiency is higher.

#### 3.2.3. Analysis of the energy for CO_2_ heating

**Fig. 7:**
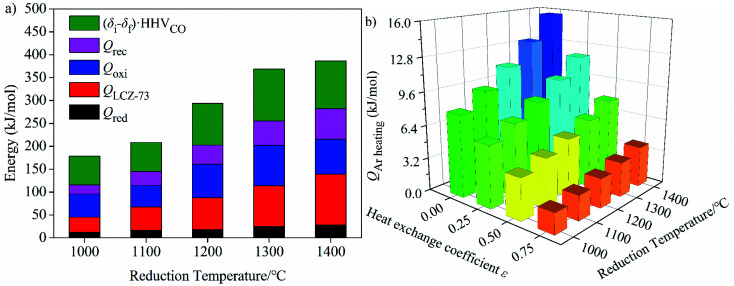
Relationship between the energy and reduction temperature during the solar two-step thermochemical CO_2_ splitting process *via* Ar purging. (a) Energy required as a function of reduction temperature, (b) energy required for Ar heating as functions of reduction temperature and heat transfer coefficient *ε*_heat exchanger_.

Fig. 7 shows the relationship between the energy of each part and reduction temperature during the solar two-step thermochemical CO_2_ splitting process *via* Ar purging. The variations of energy with reduction temperature and the relative magnitude are shown in Fig. 7(a). It can be seen that *Q*_red_, *Q*_LCZ-73_, *Q*_oxi_, *Q*_rec_ and the fuel energy generated increase with the increase of reduction temperature. The energy required for the reduction reaction is the minimum, while the energy required for the sample heating and the oxidation reaction is the maximum of the energy consumption items mentioned above. The energy required for heating Ar as functions of reduction temperature and heat exchange coefficient of heat exchanger is shown in Fig. 7(b). We can find that the energy consumed for heating Ar increases with the increase of reduction temperature, and this value at 1400 °C is about twice of that at 1000 °C. At the same temperature, the energy consumed decreases with the increase of heat exchange coefficient *ε*_heat exchanger_.

**Fig. 8:**
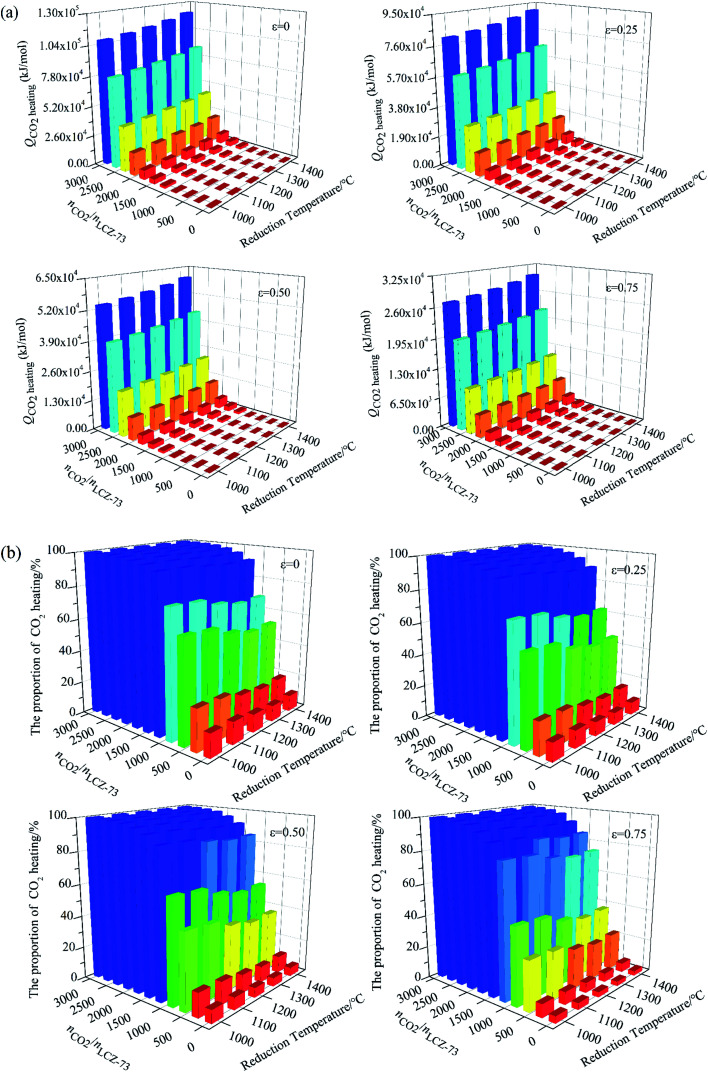
Energy for CO_2_ heating as functions of reduction temperature and molar ratio of *n*_CO_2__/*n*_LCZ-73_ under different heat recovery conditions. (a) Energy required for CO_2_ heating and (b) the proportion of energy for CO_2_ heating in total input energy as functions of reduction temperature and molar ratio of *n*_CO_2__/*n*_LCZ-73_ with different heat transfer coefficients *ε*_heat exchanger_.

Similarly, the energy consumed for CO_2_ heating with heat recovery as functions of reduction temperature and molar ratio of *n*_CO_2__/*n*_LCZ-73_ is given in Fig. 8(a). The molar ratio of *n*_CO_2__/*n*_LCZ-73_ increases as the amount of CO_2_ increases, and the energy consumed for CO_2_ heating increases significantly. When *n*_CO_2__/*n*_LCZ-73_ varies from 100 to 1000, the energy consumed increases by a corresponding multiple. Compared with the molar ratio of *n*_CO_2__/*n*_LCZ-73_, the reduction temperature has much smaller impact on the energy consumed for CO_2_ heating. The energy increases slightly with the increase of reduction temperature, but the trend is gentle. The energy for CO_2_ heating is two or three orders of magnitude higher than those in Fig. 7(a) and (b).

Finally, the proportion of energy required for CO_2_ heating in total input energy is summarized in Fig. 8(b). It can be seen that the proportion rapidly increases with the increase of *n*_CO_2__/*n*_LCZ-73_, but decreases slightly with the increase of the reduction temperature, which is mainly because the reduction temperature has little influence on the energy of CO_2_ heating, but has great impact on the total input energy. With the increase of heat exchanger coefficient, the proportion of energy consumption for CO_2_ heating gradually decreases. As the molar ratio of *n*_CO_2__/*n*_LCZ-73_ increases, the proportion of energy for CO_2_ heating in total input energy increases rapidly, resulting in lower solar-to-fuel efficiency. At 1300 °C and *ε*_heat exchanger_ = 0.5, when *n*_CO_2__/*n*_LCZ-73_ = 0.5, the proportion of CO_2_ heating is only 5.35%, while, when *n*_CO_2__/*n*_LCZ-73_ = 50, the proportion reaches nearly 85.0%. When the amount of CO_2_ is further increased, this proportion reaches 95.8% as the ratio of *n*_CO_2__/*n*_LCZ-73_ increased to 200. This also reveals the reason why the current thermochemical fuel research is less efficient.

#### 3.2.4. The effect of CO_2_ flow rates on solar-to-fuel efficiency

**Fig. 9:**
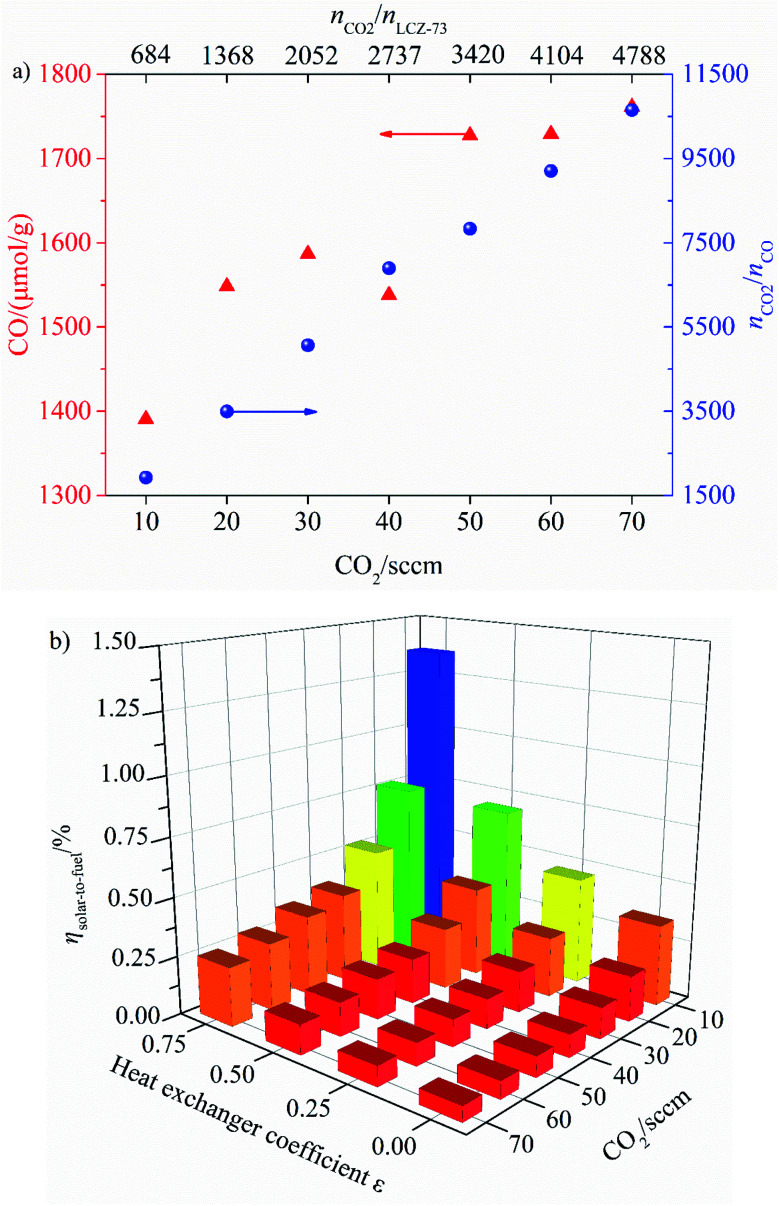
CO yield and solar-to-fuel efficiency as functions of CO_2_ flow rate.

In order to explore the thermodynamic properties of perovskite LCZ-73 based thermochemical CO_2_ splitting under different CO_2_ content atmospheres, experimental tests are carried out with different CO_2_ content in Ar. Approximately 10 mg LCZ-73 powder is used for each test and the sample is heated to 1300 °C in Ar (80 sccm) with the ramp of 10 °C min^−1^ and maintained for 60 min. Then the sample is cooled down to 800 °C for oxidation with the ramp of 20 °C min^−1^ and maintained for 60 min in the atmosphere of 10–70 sccm CO_2_ balanced with Ar, the corresponding ratio of *n*_CO_2__/*n*_LCZ-73_ varies from 684 to 4788, the total gas flow is maintained at 80 sccm. The CO yield and solar-to-fuel efficiency as functions of CO_2_ flow rate are obtained and calculated based on the thermodynamic model mentioned above. The details are shown in Fig. 9.

As shown, the effects of CO_2_ flow rate on CO yield and solar-to-fuel efficiency at various heat exchanger coefficient *ε*_heat exchanger_ are analyzed. It can be clearly seen from Fig. 9(a) that the yield of CO increases gradually with the increase of CO_2_ flow rate, and the high CO_2_ concentration is favorable for the oxidation reaction. At the same time, the ratio of *n*_CO_2__/*n*_CO_ changes monotonically with the increase of CO_2_ flow rate. When the CO_2_ flow rate in the oxidation step is 10 sccm, *n*_CO_2__/*n*_LCZ-73_ = 684, *n*_CO_2__/*n*_CO_ = 1926, and the same value is 10 648 when the CO_2_ flow rate is 70 sccm at *n*_CO_2__/*n*_LCZ-73_ = 4788, the latter represents an increase of nearly 27% in CO yield over the former. However, it is also noted that solar-to-fuel efficiency is negatively correlated with the increase of CO_2_ flow rate in Fig. 9(b). Although CO yield is low at low CO_2_ flow rate, solar-to-fuel efficiency is high. In order to prove that this material can still split CO_2_ under the condition of lower molar ratios, further experiments would be needed for verification, such as the CO_2_ flow rate is less than 10 sccm (*n*_CO_2__/*n*_LCZ-73_ < 684). It should be noted that an excessively low CO_2_ flow rate may impose higher requirements on the experimental devices in actual operation. Considering the effect of heat transfer coefficient, solar-to-fuel efficiency increases with the increase of heat transfer coefficient at the same CO_2_ flow rate. The maximum solar-to-fuel efficiencies of 1.36% and 0.35% are achieved with 75% heat recovery and without heat recovery with the CO_2_ flow rate of 10 sccm (*n*_CO_2__/*n*_LCZ-73_ = 684). However, the solar-to-fuel efficiencies under the same heat exchange conditions are only 0.25% and 0.06% with the CO_2_ flow rate of 70 sccm.

## 4. Conclusions

This work performs the performance evaluation towards the doped perovskite LaCo_0.7_Zr_0.3_O_3_ (LCZ-73) based thermochemical CO_2_ splitting process thermodynamically *via* the experimentally derived parameters for the first time. The impacts of vacuum pump and vacuum pump combined with inert gas purge to reduce oxygen partial pressure and CO_2_ heating on the performance parameter *η*_solar-to-fuel_ have been analyzed with the consideration of gas–gas, gas–solid phase heat recuperation. The energy cost of CO_2_ heating during the redox process is underscored. At the oxygen partial pressure of 10^−5^ bar, non-stoichiometric oxygen *δ* increases by more than 3 times as the reduction temperature increases from 1000 °C to 1300 °C. However, the deviation of *δ* is negligible between 1300 °C and 1400 °C. The reaction enthalpy ranges from 60 to 130 kJ mol^−1^ corresponding to *δ* = 0.05–0.40. Comparing *η*_solar-to-fuel_*via* vacuum pump with the case of vacuum pump combined with inert gas purging, the efficiencies of 0.39% and 0.1% can be achieved with 75% and without heat recovery under experimental conditions, respectively. Based on the energy analysis, the energy cost of CO_2_ heating took up the bulk of energy consumption of the thermodynamic process as the *n*_CO_2__/*n*_LCZ-73_ increases, which is two or three orders of magnitude higher than other heating items. Compared with higher temperature conditions, *n*_CO_2__/*n*_LCZ-73_ at lower temperature requires more demanding conditions for the aim of commercialization. Finally, the ability of the perovskite LCZ-73 to split CO_2_ and thermochemical performance were tested under different CO_2_ flow rates. The results show that high CO_2_ flow rate is conductive to the production of CO, but at the cost of low *η*_solar-to-fuel_. The maximum solar-to-fuel efficiencies of 1.36% and 0.35% are achieved with 75% heat recovery and without heat recovery with the CO_2_ flow rate of 10 sccm. However, values under the same heat exchange conditions are only 0.25% and 0.06% with the CO_2_ flow rate of 70 sccm. On the premise of meeting the requirements of the experimental device, further experiments are needed to verify the ability of the perovskite to split CO_2_ at lower CO_2_ flow rate (*e.g.*, less than 10 sccm, *n*_CO_2__/*n*_LCZ-73_ < 684).

## Nomenclature

*M*
: The molar mass of perovskite, g mol^−1^
*m*
: Mass of the matter, g
*R̄*
: Universal molar gas constant, 8.314 J mol^−1^ K^−1^
*T*
: Temperature, K
*K*
: The equilibrium constant
*P*
: Pressure, atm
*Q*
: The energy required or recovered for each process, J mol^−1^
*C̄*_p_
: The specific molar heat capacity, J mol^−1^ K^−1^
*n*
: Amount of substance, mol
HHV: Higher heating value, J mol^−1^
*C*
: Solar concentration ratio, suns
*I*
: 1000 W m^−2^
*n*_CO_2__/*n*_LCZ-73_: Molar ratio of CO_2_ to perovskite LaCo_0.7_Zr_0.3_O_3_

### Greek symbols

Δ*m*: Mass change, g
δ
: Nonstoichiometric oxygen
Δ*G*^Θ^: Standard molar Gibbs free energy, J mol^−1^
Δ*H*^Θ^_red_: Standard molar enthalpy for reduction, J mol^−1^
Δ*S*^Θ^_red_: Standard molar entropy for reduction, J mol^−1^
Δ*δ*: Non-stoichiometry change
Δ*Q*: The energy change in a process, J mol^−1^
Δ*T*: Temperature swing, K
*η*_S→W_
: Solar energy to vacuum pumping conversion efficiency
*ε*_heat exchanger_
: The heat exchange coefficient of the heat exchanger
*ε*_rec_
: The effectiveness of heat recovery, 0.6
*η*_abs_
: Heat absorption efficiency of solar reactor
σ
: Stefan–Boltzmann constant, 5.67 × 10^−8^ W m^−2^ K^−4^
*η*_solar-tio-fuel_
: Solar to fuel conversion efficiency

### Subscripts

O: Oxygen atom
O_2_: Oxygen gas
red: Reduction
oxi: Oxidation
i: The state of non-stoichiometric oxygen after reduction
f: The state of non-stoichiometric oxygen after oxidation
pump: Vacuum pump
0: The atmospheric pressure, 1 atm
Ar heating: Preheat the sweeping gas Ar
amb: Ambient condition
CO_2_: Carbon dioxide
CO: Carbon monoxide
rec: Recovery
CO_2_ heating: Preheat the CO_2_ gas
abs: Absorption

### Superscripts

Θ: Standard condition at *T* and *P*_atm_

### Abbreviations

LCZ-73: LaCo_0.7_Zr_0.3_O_3_

## Conflicts of interest

There are no conflicts to declare.

## Supplementary Material
